# Book Reviews

**Published:** 1875-05-15

**Authors:** 


THE HISTORY OF THE PHILADELPHIA SCHOOL OF ANATOMY, AND ITS RELATIONS TO MEDICAL TEACHING. A Lecture, delivered March 1st, 1875, at its dissolution, by Wm. B. Keen, M.D., lecturer on Anatomy, etc, Philadelphia: J. B. Lippincott & Co., 1875.

This is a very well written and interesting lecture, well calculated to illustrate the importance of a thorough study of anatomy. It is in the form of a neatly printed pamphlet of thirty-two pages.

---

A PRACTICAL TREATISE ON THE MEDICAL AND SURGICAL USES OF ELECTRICITY. By Geo. M. Beard, M.D., etc., and A. D. Rockwell, M.D. New York: Wm. Wood & Co. Chicago: for sale by Jansen, McClurg & Co. Price, $6.25.

The authors of this work have expended a large amount of time and labor upon this second edition. It has been thoroughly revised and in grêat part re-written, as well as considerably enlarged. We cannot better indicate the character and extent of the revisions made than by quoting from the author's preface:

"A few weeks after the publication of the first edition of this work, in 1871, we were informed by the publishers that a new edition would be called for. From that time to the present moment much force has been expended on the thorough revision of the work in all its departments. As much time and toil, it is safe to say, have been given to this edition as to the first; and the work, as it now stands, represents our accumulated and thoroughly sifted experience from our entrance upon this specialty, as well as a full and exhaustive resume of all that has been accomplished by other authorities everywhere.

"The use of general faradization as a constitutional tonic in a wide variety of affections is now well established, and the effects that we have claimed for it have been confirmed in full detail by competent observers at home and abroad. This method of using electricity has also attained a wide popularity, and its introduction into therapeutics may be said to have marked a radical and important advance.

"The section on Electro-physics is much enlarged. Observation has convinced us that the one great defect in those who practice electro-therapeutics is ignorance of the physical relations of electricity. From this source flow at least half the blunders, discouragements, and ill-success that novices in this branch so painfully experience. The undulatory theory of the electrical force that is adopted in this edition is, so far as can now be seen, consistent and harmonious, and it explains better than any other theory the varied and complex phenomena of electro-physiology and electro-therapeutics.

"Electro-physiology is largely re-written and considerably enlarged. It includes a large number of our own experiments, mostly made during the ast three years, as well as a compact resume of all the more recent studies in this branch by European and American observers. The general relation of electro-physiology to electro-therapeutics has been brought into prominence at every point.

"The method of *central galvanization* that we have systematized and introduced to the profession, since the publication of the first edition, is here described and illustrated in full detail. The great practical advantages of this method of galvanization over localized galvanization of the nerve-centres—and in many cases over general faradization — are already well understood by many of our leading electro-therapeutists.

"There are now introduced into science six methods of using electricity for the treatment of disease : localized faradization and localized galvanization, general faradization, central galvanization, and, in electro-surgery, electrolysis, and galvano-cautery.

"In the chapter on Apparatus we have endeavored to represent with fairness and impartiality the best workmanship and the most recent improvements. The fact of the superiority of continuous over separate-coil Faradic-machines in the treatment of sensitive patients is here for the first time brought out and emphasized.

"A new chapter on General Suggestions has been added, in which the attempt has been made to answer in detail the various practical queries that so annoy the beginner in electro-therapeutics.

"In the section on Electro-surgery the principles of galvano-cautery, of ordinary electrolysis, and of the method of electrolysis of the base have been described and illustrated, and in the clinical portions all varieties of results have been presented from a very large experience in this department, so that one may learn both what can be done and what cannot be done by electricity in surgical diseases.

"In the clinical part of electro-medicine a number of entirely new chapters have been added, and all of the chapters have been re-cast. The number of cases has been increased nearly twofold, the failures and successes being fairly represented.

"We may call especial mention to the chapters on Diseases of the Skin, wherein, besides many other cases, are detailed the remarkable results of central galvanization in chronic eczema and prurigo, and to the chapter on Diseases of Children, in which are recorded the results of experiments in the treatment of whooping-cough, marasmus, and debility, and also the fact of the remarkable tolerance of childhood to electricity. Since the publication of the first edition a number of excellent works on nervous diseases have appeared, and for that reason, as well as for lack of space, the systematic remarks on certain diseases have, in this edition, been mostly omitted, save some special points wherein our views differ from those generally adopted."

THE TREATMENT OF NERVOUS DISEASES BY ELECTRICITY. By Dr. Friedrich Fieber. Translated from the German. New York: G. P. Putnam's Sons. For sale by Jansen, McClurg & Co., Chicago. 12mo. 64 pp. Price, 75c.

This little monograph contains a condensed review of the present extent of electrical treatment in nervous diseases, with indications for its employment. Any attempt to cover so wide a field in such extremely brief space must necessarily, however, be unsatisfactory, from its too general and superficial character. The ideas and doctrines advanced are nevertheless good and practical as far as they go.

---

A HAND-BOOK OF THERAPEUTICS. By Sidney Rivyer, M.D. Fourth edition. New York: Wm. Wood & Co. For sale by Jansen, McClurg & Co., Chicago. Price, $4.25.

This work has been most favorably received by the profession in its former editions, and needs no extended review.

---

ZIEMSSEN'S CYCLOPÆDIA. — The publishers announce that this work will not be sold in *separate volumes*, but only in complete sets. Those desiring the complete work are therefore cautioned against purchasing any odd volumes that may be offered for sale.

---

ANATOMICAL PREPARATIONS,

Including Skeletons, and the Separate Bones of the Human Subject, can be obtained at reasonable prices, of

E. H. SARGENT,
Dealer in Surgical Instruments,
CHICAGO.

